# Predictors of zoonotic potential in helminths

**DOI:** 10.1098/rstb.2020.0356

**Published:** 2021-11-08

**Authors:** Ania A. Majewska, Tao Huang, Barbara Han, John M. Drake

**Affiliations:** ^1^ Odum School of Ecology and the Center for Ecology of Infectious Diseases, University of Georgia, Athens, GA, USA; ^2^ Biology Department, Emory University, Atlanta, GA, USA; ^3^ Cary Institute of Ecosystem Studies, Millbrook, NY, USA; ^4^ Ecology, Evolution, and Behavior, Boise State University, Boise, ID, USA

**Keywords:** macroparasite, parasite trait, zoonosis, pet, intermediate host

## Abstract

Helminths are parasites that cause disease at considerable cost to public health and present a risk for emergence as novel human infections. Although recent research has elucidated characteristics conferring a propensity to emergence in other parasite groups (e.g. viruses), the understanding of factors associated with zoonotic potential in helminths remains poor. We applied an investigator-directed learning algorithm to a global dataset of mammal helminth traits to identify factors contributing to spillover of helminths from wild animal hosts into humans. We characterized parasite traits that distinguish between zoonotic and non-zoonotic species with 91% accuracy. Results suggest that helminth traits relating to transmission (e.g. definitive and intermediate hosts) and geography (e.g. distribution) are more important to discriminating zoonotic from non-zoonotic species than morphological or epidemiological traits. Whether or not a helminth causes infection in companion animals (cats and dogs) is the most important predictor of propensity to cause human infection. Finally, we identified helminth species with high modelled propensity to cause zoonosis (over 70%) that have not previously been considered to be of risk. This work highlights the importance of prioritizing studies on the transmission of helminths that infect pets and points to the risks incurred by close associations with these animals.

This article is part of the theme issue ‘Infectious disease macroecology: parasite diversity and dynamics across the globe’.

## 1. Introduction

Understanding the factors that contribute to the emergence of novel infectious diseases is a central concern to global public health [1]. Since most outbreaks of novel pathogens among humans are owing to spillover from animal hosts (wild and domestic) [2–4], identifying factors associated with the propensity for transmission to humans is of high priority. Research in this area is particularly urgent because the rate of human–wildlife contacts is increasing with changes to natural landscapes and global climate [5], providing ample opportunities for human exposure to novel hosts and pathogens [6,7]. Further, parasite sharing between domestic and wild animals provides another interface for transmission to humans [8]. Identifying species that are potentially parasitic or pathogenic in humans (i.e. those with high *zoonotic potential*) would enhance our understanding of the factors underpinning spillover transmission from animal reservoirs, and enable pre-emptive approaches to disease control.

One approach to evaluating zoonotic potential is to analyse pathogen and host traits (e.g. [9]). Particularly, features distinguishing zoonotic from non-zoonotic parasites and their reservoir host species can be used to predict which host species are most likely to present high risk of zoonotic exposure to people [10]. For example, work by Han *et al*. [11] identified ‘fast’ life-history strategy (short-lived, short generation time) as a key predictor of the rodent species most likely to be reservoirs of novel zoonotic pathogens. The same approach can be applied to predict characteristics of zoonotic parasites: trait analysis of zoonotic viruses revealed that viruses which can replicate in cytoplasm are more likely to infect humans [12]. Similarly, viruses which infect non-human primates are more likely to spread between humans [13]. Patterns in genome sequences of viruses have also yielded predictions about which hosts are likely to be reservoirs of zoonoses and which arthropods are likely to be their vectors [14]. These findings are of scientific interest concerning theoretical debates about why some parasite species are more prone to spillover than others [15–17].

Parasitic helminths are a group of parasites that remains poorly studied in comparison to viruses and bacteria, but may pose considerable future risk to humans. Helminths are macroparasites, typically tapeworms (cestodes), roundworms (nematodes) or flatworms (trematodes), and are primarily known for chronic infections of the gastrointestinal tract, although helminths can infect nearly all human tissues [18]. A handful of helminth species cause massive disease burden. Specifically, schistosomiasis (*Schistosoma* spp.), soil-transmitted helminthiasis (e.g. *Necator americanus* and *Ascariasis lumbricoides*) and filariasis (e.g. *Wuchereria bancrofti*) are thought to infect more than one-quarter of the human population [19,20]. Helminths are also known to be vectors for other zoonoses, such as the fever-causing bacteria *Neorickettsia sennestu* transmitted by a trematode ingested via raw fish consumption [21]; although helminth vectoring is relatively poorly understood [22]. Human–helminth associations have ancient origins (reviewed in [23]), but the relatively recent domestication of animals for food and companionship significantly increased the number of parasites shared between humans and (domesticated) animals [24]. The agricultural revolution and associated practices, such as storage of crops in granaries, probably created new links between humans and wildlife, providing additional opportunities for helminth species to infect human hosts [25]. Indeed, humans, domesticated animals and wildlife share a number of parasitic helminths [8,24] and zoonotic helminths continue to emerge within human populations, a process that is expected to accelerate with the global trade of livestock, climate change and growth in the demand for animal protein for human consumption [26].

Helminths commonly have complex life cycles that rely on one or more intermediate hosts [27,28]. These intermediate hosts are necessary for the development of juvenile life stages (eggs and larvae) and transmission to the definitive host, where the animal matures, reproduces and produces propagules [29]. Intermediate hosts include a wide range of aquatic, terrestrial, wild and domesticated animals [29], yet it is unknown how intermediate host identities are linked to the risk of helminthiasis in humans. In addition, transmission may occur directly (i.e. trophically, vertically) and/or indirectly (i.e. via environment or arthropod vector). From a public health perspective, most chronic infections are caused by soil-transmitted helminths [30]; however, the transmission modes of most zoonotic helminths have not previously been reported. Thus, identifying helminth biological and ecological traits that are linked to zoonosis can help to improve our understanding of the factors that drive zoonotic potential in helminths and to better manage the risk of transmission to humans.

In addition to intrinsic biological and ecological traits such as identity of definitive and intermediate hosts, transmission to humans also may be influenced by socio-economic factors specific to regions where the parasites are found. Currently, most helminth infections in humans are found in low- and middle-income countries of the tropics [30,31], where disease prevention and healthcare infrastructure vary greatly. Numerous parasitic worms such as hookworms (genera *Ancylostoma* and *Necator*) are considered neglected tropical diseases which could be eliminated with sufficient drug administration and effective interventions [31]. Further, given the generally high animal biodiversity of tropical regions as well high estimated cumulative community-level association risk (total risk for all wild species in local pool) of wildlife carrying a zoonotic helminth [8], it also may be that there are more host species of potential zoonoses in this part of the world [32]. Yet, mammal hosts in temperate regions also show high risk of harbouring zoonotic helminth species [8,10]. Thus, we conjectured that geographical characteristics of helminths might be important for predicting the probability that a species might infect humans. Despite the high variation in medical, educational and economic burden of human helminth infections worldwide [31], how the different epidemiological and geographical factors relate to helminth zoonotic potential has been unclear.

We investigated which traits of helminths are predictors of disease in humans. We compiled a global dataset from existing databases and the published literature on more than 700 mammal helminth parasite species to examine the frequency of biological (transmission, morphology), epidemiological and geographical traits. We used boosted regression trees (BRT), an ensemble learning technique, to navigate the high dimensionality of these data. These and similar machine learning methods are rapidly developing approaches that can be applied to heterogeneous covariates and are typically robust to nonlinear interactions hidden in the data [33,34]. Among over 70 variables, our machine learning approach identified key trait patterns predicting helminth zoonosis. Specifically, whether a helminth species is zoonotic was best predicted by three characteristics: (i) whether one of the hosts is a companion animal (i.e. dog, cat), (ii) whether an intermediate host is a fish (member of Chordata phylum), and (iii) the number of unique locations (number of unique occurrence points based on latitude and longitude) in which the helminth species has been detected. More generally, this study adds to the growing body of literature used to inform strategies for preventing helminth infection and mitigating risk of novel zoonoses.

## 2. Methods

### (a) Data compilation

We used the Global Mammal Parasite Database (GMPD) [35], which consists of over 700 species of helminths, representing three main phyla (Acanthocephala, Nematoda and Platyhelminthes) of parasitic helminths that infect wild mammals. We note that GMPD is not complete and does not contain all zoonotic helminths. However, an advantage of using the GMPD is that the records were collected specifically for mammals in a standardized way, namely via reproducible search criteria. Given that most emerging diseases originate from mammals [36], a mammal-focused dataset and analysis is well suited to identifying zoonotic risk factors among mammal-borne helminths. For each helminth species, we searched primary literature for evidence of human infection originating from animal hosts to assign a binary response indicating whether or not the helminth species is zoonotic. We acquired morphological information of adults and eggs from Benesh *et al*. [37] and Dallas *et al*. [38], both of which gathered information from the literature. To fill in gaps, we followed Dallas *et al*. [38] and searched for missing morphological information from veterinary and parasitology references (e.g. Taylor *et al*. [39]), taxonomy references [29,40] and primary literature. We extracted the minimum, mean and maximum body length and width (in millimetres) of adult helminths from the descriptions of each parasite species. Specifically, we compiled records of male and female body sizes when sex was indicated and otherwise noted body size for adult worms. We also extracted the minimum, mean and maximum egg length and width (in millimetres). We recorded the site of infection in the definitive host body, noting the system as integumentary, muscular, nervous, digestive, circulatory, respiratory and reproductive system, when it was provided. From the site of infection, we derived binary variables to indicate whether a given species infected each of the body systems.

We supplemented transmission information within the above references by extracting the following: common name(s) of definitive and intermediate hosts, whether the species has a free-living propagule stage (a binary variable), and if so, the stage of the free-living propagule as egg, larva or both (as can occur in species that pass through more than one intermediate host), and the medium in which free-living stage(s) persist (soil, water or both). We used the common names of intermediate hosts to note the class or phyla to which the intermediate animal host belongs, whether any of the host (definitive or intermediate) are domesticated animals (livestock and pets), or companion pet animals (predominantly cats and dogs). Livestock animals included poultry (chicken, turkey, geese, duck), cattle (buffalo, horse, yak, zebu) and others (alpaca, goat, camel, pig, sheep, llama). For each species, we noted the transmission mode(s) to the definitive host as vertical (from parent to offspring), environmental (propagules acquired from the soil, water or both), vector (via biting arthropod) or trophic (via consumption of intermediate host). References used to compile traits for each helminth species are listed in the dataset (https://doi.org/10.6084/m9.figshare.14591664) [41].

The GMPD provides geographical coordinates for each helminth species, which we augmented with host–helminth occurrence data from the London Natural History Museum (LNHM) [42], a freely accessible database that can be systematically explored with web-scraping tools such as R package helminthR [43]. Coordinates in the GMPD are from reported study site coordinates, or centroids of the reported study area [35]. Helminth occurrences in LNHM are georeferenced as centroids to the country or state (for the USA) level. In several instances, coordinates were not provided by the databases, which we then georeferenced based on the location name using the *geocode* function (package ggmap; [44]). Some location names were vague, such as the portion of a continent (e.g. southern South America) or body of water (e.g. southwest Atlantic), which we did not georeference. Next, based on the occurrence points of each species, we calculated the number of unique locations and latitudinal range (minimum and maximum), assigned a binary variable to indicate whether the species occurrences fall within the tropical latitudes (between 23° 27′ N and 23°27′ S), and quantified the number of occurrences within tropical latitudes. We note that the number of unique locations reflects geographical distribution and sampling effort (for further exploration of sampling bias, see the electronic supplementary material). From occurrence data, we also calculated the number of countries, terrestrial ecoregions of the world (as defined by Olson *et al*. [45]), and terrestrial zoogeographic realms (as defined by Holt *et al*. [46]) from which each helminth species has been reported. Further, following Byers *et al*. [47], we calculated range size for each helminth species as the total area of the ecoregions in which the species has been found. Finally, we obtained the mean gross domestic product (GDP) and human population size from the World Bank (via package wbstats in R [48]) for countries in which the species has been documented for the most recent year data are reported. Our final dataset consisted of 737 globally distributed helminth species (electronic supplementary material, figure S1) and 73 trait variables describing helminth species that we included in our analyses. We classified the traits into one of four categories: transmission, epidemiological, morphological or geographical traits (table 1). For full descriptions of each variable and additional details on variable compilation, see the electronic supplementary material, tables S1 and S2.

**Table 1.** Top 15 most important variables used to predict helminth zoonoses status. (Colours of the rows correspond to the four trait categories: geographical traits are in pink, transmission traits are in green, morphological traits are in blue and epidemiological traits are in orange. Colour scheme also applies to figure 3.)

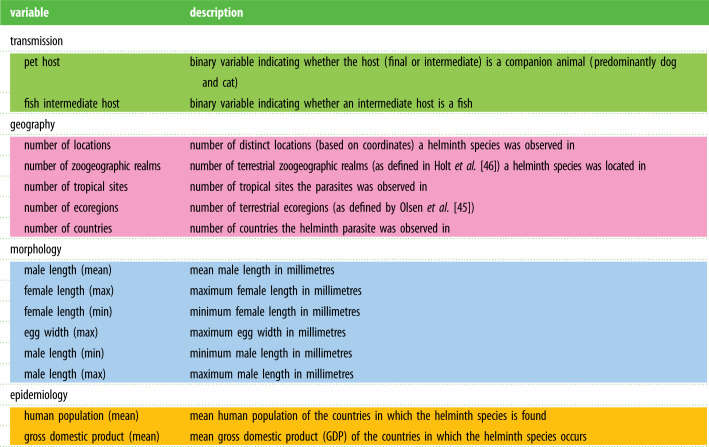

### (b) Predictive model

**Figure 1.**
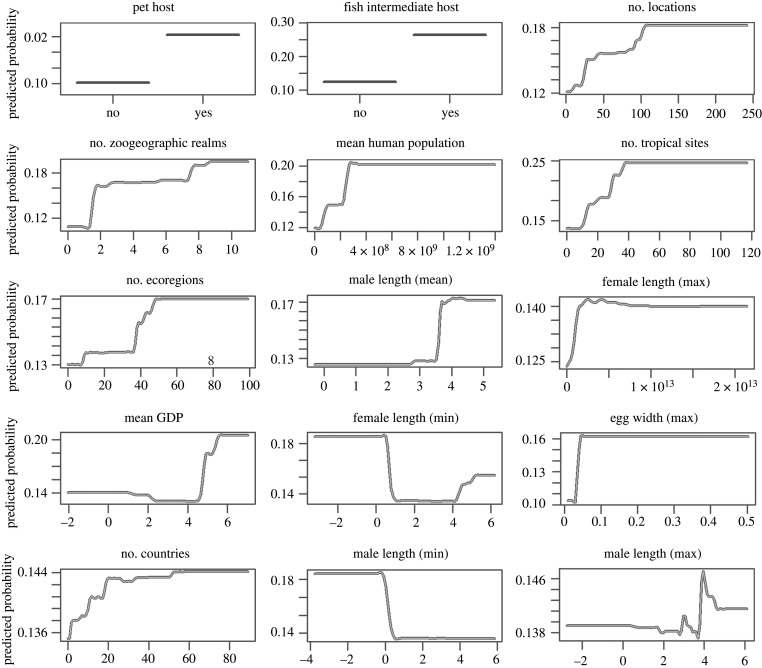
Partial dependence plots for top 15 most important variables. Plots are based on permutations of the primary BRT model that included 73 variables. Importance of the variables is ordered from left to right, then top to bottom. Black lines represent the median predicted probability, while shaded regions represent the corresponding 95% confidence interval across 100 permutations of the model.

**Figure 2.**
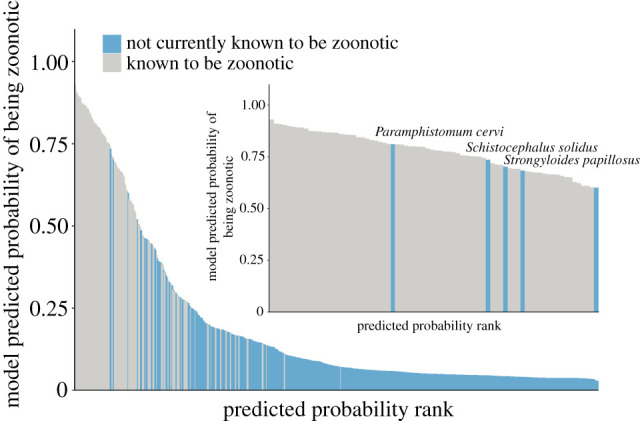
Predicted zoonotic helminth risk index. Average model-predicted probability of being zoonotic as ranked by the primary BRT model. Blue bars represent species not known to be transmissible to humans from wildlife and grey bars are species known to be transmissible to humans from wild hosts and are confirmed by the model to be zoonotic. Inset: zoonoosis risk of helminth species with model-predicted probabilities greater than 70%. Names of top three species not currently known to be zoonotic appear above the bars and include *Paramphistomum cervi, Schistocephalus solidus* and *Strongyloides papillosus* (in descending order). (Online version in colour.)

We used BRT, a regression approach that permits missing data, variable interactions, collinearity and nonlinear relationships between the response and explanatory variables, which can be of mixed types [33,49]. We fit a logistic-like predictive model with the zoonotic status of the helminths (0, not zoonotic; 1, zoonotic) as the response variable and the 73 traits as explanatory variables. Prior to analysis, we log transformed body size variables and range size, which were right skewed. We randomly selected 80% of the data as the training set and reserved 20% for testing. BRT were trained using the gbm package in R [50] with Bernoulli distributed error. We ran permutations of the model with different learning rates (1 × 10^−5^ to 1 × 10^−2^) and tree depths (1–3) using the training set to identify optimal learning parameters yielding the highest predictive performance (see the electronic supplementary material, figure S2). The learning conditions that maximized accuracy as assessed by the model AUC (area under the receiver operating characteristic curve) included setting the maximum number of trees to 50 000, a learning rate of 0.001 and an interaction depth of 3. We acquired output on the relative influence score, which we refer to as relative importance, for each predictor variable, which are computed via permutation procedures across all the trees generated using Friedman's algorithm [49]. Briefly, relative influence is computed as the average improvement in the mean squared error at tree splits across all the trees in which a given variable is present. We also present partial dependence plots showing the marginal effect of each variable on the predicted outcome of the primary model [33,49] (figure 1). Based on the results of the primary model, we ranked helminth species by their predicted probability of being transmissible to humans (figure 2).

**Figure 3.**
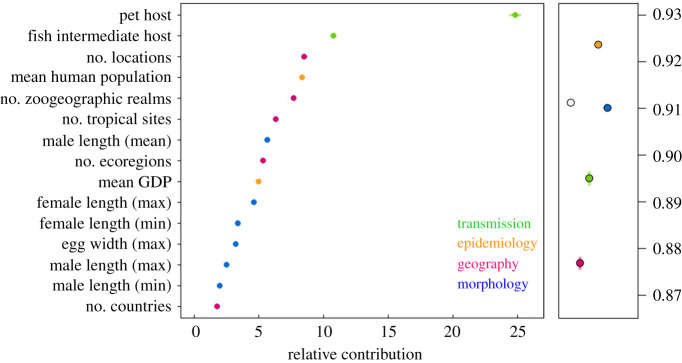
Variable importance values by permutation, averaged over 100 models trained on all four categories of traits (left panel), show relative importance of transmission traits (green), epidemiological traits (orange), geographical traits (maroon) and morphological traits (blue). Average model accuracy for each submodel trained on all four trait categories (white symbol), all trait categories except: morphological traits (blue), epidemiological traits (orange), transmission traits (green) or geographical traits (maroon). Error bars represent the standard deviation from 100 model permutations. (Online version in colour.)

Finally, to explore the importance of trait categories, we repeated the above analysis using only the top 15 most important variables predicted by the primary model trained on all 73 variables, and permuted the model 100 times. We created additional submodels, also permuted 100 times, with each of the four trait categories (transmission, epidemiology, morphology, geography; table 1) excluded and explored the resulting model performance in predicting zoonotic versus non-zoonotic species (figure 3; electronic supplementary material, figure S4). Model performance was assessed via model AUC score. All analyses were performed in R [51].

## 3. Results

We examined 737 globally distributed helminth species of which 137 are known to infect humans. Our boosted regression ensemble of models trained on 73 helminth traits distinguished zoonotic versus non-zoonotic species with test set withheld with 88% accuracy (AUC = 0.88) and identified several predictors of zoonotic helminths (figure 1). The ensemble of models predicted the testing set of the data with 91% accuracy (AUC = 0.91). Of the 73 traits, 57 traits had non-zero influence (for the relative influence values of all 73 variables, see the electronic supplementary material, table S2). The most important variable for accurately predicting zoonotic helminths was whether the helminth species is known to infect a companion animal, followed by whether fishes serve as intermediate hosts, and the number of locations in which the helminth species has been documented. The fourth and fifth most important traits predicting zoonotic status in helminths related to the size of terrestrial zoogeographic regions observed for each helminth species (figure 1). Generally, the most important traits were related to geography and transmission, while epidemiological and morphological traits were least important (electronic supplementary material, table S2).

While not currently known to cause human infection, BRT models identified three mammal-borne helminth species as likely to be zoonotic with greater than 70% probability (figure 2) (in descending order): *Paramphistomum cervi, Schistocephalus solidus* and *Strongyloides papillosus.* (For a full list of helminth species and the predicted probabilities, see the electronic supplementary material, table S5.)

Additional ensembles of BRT models restricted to the top 15 most important variables (as identified by the primary models with 73 traits included, see figure 1) predicted the testing data with similar accuracy (AUC = 0.91 for primary models trained on all 73 traits and 15 most important variables). The restricted submodels trained on the 15 variables generally agreed on the ranking of the importance of variables with the primary models (figure 3). Submodels trained on data without one of the trait categories (i.e. leave-one-out) indicated that models trained on data without morphological traits performed slightly worse (AUC = 0.90) compared to submodels with all trait categories included (AUC = 0.91; figure 3), suggesting that including these features improved the predictive accuracy of our models. Models trained on data with epidemiological traits left out performed best (AUC = 0.92; figure 3). Finally, models trained on data without geographical traits or transmission traits performed worse than models with other categories left out (AUC = 0.88, AUC = 0.89, respectively; figure 3). In submodels, companion animal host was the most important variable, except for the submodel that excluded transmission traits (electronic supplementary material, figure S4). For the relative influence values of the variables in submodels, see the electronic supplementary material, table S4.

## 4. Discussion

Identifying pathogen traits associated with a propensity to spillover into humans is key for understanding and predicting emergence of novel human diseases originating from wildlife. We applied a machine learning algorithm to a large dataset of mammal helminths to identify characteristics distinguishing zoonotic and non-zoonotic species, and to predict which species currently classified as non-zoonotic have a high risk of spilling over to humans in the future. Our results indicate that helminths that infect companion animals (dogs and cats) and use fishes as intermediate hosts are more likely to cause human infection compared to other mammal-borne helminths. The third strongest predictor of the ability to cause human infection was the number of occurrences of helminth species, which indicates that widespread geographical distribution might provide important transmission exposure to human hosts; however, we note that this variable might also reflect sampling effort (see below). Overall, these results suggest that the zoonotic potential of helminth species is related to the identity of both definitive and intermediate hosts that come in direct and indirect contact with people, thereby providing abundant opportunities for parasite transmission. Further, our findings highlight the importance of transmission strategies in the ability of mammalian helminths to infect humans.

Particularly interesting is the predicted association between helminth zoonosis and companion animals (predominantly cats and dogs in this study). Domestic cats and dogs are hosts to numerous parasitic helminth species [39,52] and represent an important link between humans and wildlife for zoonosis [53]. Indeed, the role of cats and dogs in helminthiasis have been well documented for several parasites including the zoonotic tapeworm *Echinococcus multilocularis* [54] and roundworm *Toxocara cati* [53]. Our findings are in agreement with recent work which used network analysis of helminths, domesticated animals, wildlife and humans and showed high risk of wildlife sharing helminths with humans and dogs [8].

While many domesticated cats and dogs are free-ranging (i.e. not restricted to a single household or area), these animals are ubiquitous and tend to live near humans for provisioned food and shelter. Further, they hunt wild animals, consume animal parts (e.g. entrails) discarded by humans, and can overlap with wildlife habitat and territories [55], even in urban areas where numerous wild animals such as racoons, foxes and coyotes thrive [56,57]. The direct trophic interactions and indirect contacts dog and cats have with wildlife provide numerous opportunities for transmission of helminth parasites from wild to domestic animals, and eventually to humans. Dogs, in particular, might serve as spillover bridges between wildlife and humans owing to their sharing of parasites and contact with wildlife [58]. Additionally, the human–pet–wildlife interface has been around for centuries as it surfaced thousands of years ago with the domestication of cats 10 000 years ago and dogs 16 000 years ago [59,60]. Therefore, there has been ample opportunity for host-jumping and host-switching events from wildlife to pets and humans, a process which is expected to accelerate with the increasing size of the human population, associated companion animals and activities that impose close contact with wildlife.

Fishes (freshwater or marine) as an intermediate host was identified as the third most important trait for predicting zoonosis. This finding is not surprising as fishes are well-documented intermediate hosts to non-zoonotic parasitic worms that inflict humans [61]. One of the best-known examples of zoonotic parasites transmitted by fishes is nematode *Anisakis simplex,* which have a complex life cycle with marine mammals as definitive host and high incidence among human populations that eat raw fish [62]. Fish-borne helminths are transmitted via consumption of raw, undercooked or improperly preserved fish [63] and, therefore, fishes represent an important direct trophic link between humans and wildlife. While wild fishes are a well-known source of parasitic helminths [61,64], recent work indicates that farmed fishes are also linked to zoonosis [65,66]. Parasitic worm infections stemming from fish ingestion are increasing, probably owing to the significant increase in demand for fish meat associated with changes in dietary habits and population growth [67]. Our finding elucidates fishes as a key group of intermediate hosts linked to helminthiasis and the importance of monitoring fishes intended for human consumption for parasitic worms to prevent and control zoonosis.

We also identified several geographical traits as important to predicting zoonotic helminths. Specifically, the number of unique locations around the world, the number of zoological realms in which helminths have been found and the number of locations within the tropics were relatively important predictors. Overall, these findings suggest that parasitic helminths of mammals that are geographically widespread and persist in a range of habitat types are also more likely to be zoonotic than their more ecological specialized counterparts, possibly owing to their ability to persist in different environmental conditions and exposure to humans in varying environments.

It is important to note that study effort (and attendant bias) is probably interwoven through several traits we included in this study. Particularly, the number of unique record locations might not only capture distribution but also number of samples and, therefore, sampling effort. Indeed, previous work shows that variation in sampling effort among parasitic species can predict the number of localities in which the species are documented [68]. Companion animal (pet host) trait might also reflect disproportionate study effort, given the high access and relative ease of sampling. Furthermore, veterinary diagnostics (e.g. faecal floats, snap tests) more frequently performed on companion animals in high-income countries might lead to higher discovery rate of helminth species in these places. We found that submodels which included or excluded the number of occurrences resulted in companion animal (pet host) remaining the most important predictor of zoonotic status among the helminths, lending some assurance of the strong statistical association between zoonotic status and pet host despite the influence of sampling effort in helminth data.

Our model found several helminth species that are currently not known to infect humans to have high estimated probability (70% or higher) of causing zoonosis. The helminth species with highest probability of causing human infection was a flatworm, *P. cervi,* followed by *Sc. solidus,* and *St. papillosus. Paramphistomum cervi* is environmentally transmitted and requires a snail intermediate host that is accidentally ingested by wild mammals and livestock ruminants (e.g. sheep and cattle), the definitive hosts [69]. Given that livestock can share species of gastrointestinal helminths with farmers [70], and that transmission to humans via ingestion of snails (intentionally or otherwise) has been demonstrated in rat lungworm (*Angiostrongylus cantonensis*) [71], *P. cervi* may be a likely candidate for spillover to humans. On the other hand, the flatworm *Sc. solidus* infects copepods, fishes and fish-eating water birds [72], all of which have the potential to provide trophic transmission to human host. *Strongyloides papillosus* also appears likely to have the pathway to directly infect humans since it infects livestock and has a free-living generation that can persist in the environment [73]. Identifying these three species of helminths and their traits serves as an initial step in focusing efforts on surveillance and empirical work investigating the zoonotic potential of these species.

In conclusion, we focused our study on parasitic helminth traits and used BRT to quantify how the different transmission, geographical, morphological and epidemiological factors relate to helminths' zoonotic potential. Our work suggests that helminths found in cats and dogs are more likely to infect humans, and that consumption of fishes by humans may pose a greater risk of spillover. While our study examined over 700 helminth species, many more parasitic worms are found in wildlife, and most are poorly described with little known about their life cycles [74]. Key life cycle details, such as intermediate host(s), are often assumed based on relation to better-studied species in the same genus. Large gaps in our understanding of life cycles and transmission dynamics exist for most parasitic worms, including those known to infect humans. Experimental infection work is largely lacking, while detailed studies of life cycles are no longer common [74] as molecular studies have eclipsed traditional experimental biology. Despite these knowledge gaps, the machine learning approach we took point to key insights about zoonotic helminths. In particular, our results highlight the importance of the interface between wildlife, companion animals and humans in determining the risk of parasitic worm infections, which continue to cause significant disease burden in developing countries [75], where free-ranging dogs and cats are generally not treated for parasites on a regular basis and will probably continue to serve as a source of novel helminthiases.

## Data accessibility

Dataset and accompanying R code for analyses are available on figshare: https://doi.org/10.6084/m9.figshare.14591664 [41].
